# Development and validation of a neoadjuvant chemotherapy pathological complete remission model based on Reg IV expression in breast cancer tissues: a clinical retrospective study

**DOI:** 10.1007/s12282-024-01609-y

**Published:** 2024-07-08

**Authors:** Jiao-fei Wei, Fan Li, Jia-wen Lin, Zi-ang Dou, Shu-qin Li, Jun Shen

**Affiliations:** 1 Jinzhou Medical University, Jinzhou, 121001 Liaoning China; 2 Department of Breast Surgery, The First People’s Hospital of Lianyungang, No. 6 Zhenhua East Road, High-Tech Square, Lianyungang, 222002 Jiangsu Province China; 3 Lianyungang Clinical College of Nanjing Medical University, No. 6 Zhenhua East Road, High-Tech Square, Lianyungang, 222002 Jiangsu Province China

**Keywords:** Breast cancer, Neoadjuvant chemotherapy, pCR, Prediction model, Reg IV

## Abstract

**Objective:**

To develop and authenticate a neoadjuvant chemotherapy (NACT) pathological complete remission (pCR) model based on the expression of Reg IV within breast cancer tissues with the objective to provide clinical guidance for precise interventions.

**Method:**

Data relating to 104 patients undergoing NACT were collected. Variables derived from clinical information and pathological characteristics of patients were screened through logistic regression, random forest, and Xgboost methods to formulate predictive models. The validation and comparative assessment of these models were conducted to identify the optimal model, which was then visualized and tested.

**Result:**

Following the screening of variables and the establishment of multiple models based on these variables, comparative analyses were conducted using receiver operating characteristic (ROC) curves, calibration curves, as well as net reclassification improvement (NRI) and integrated discrimination improvement (IDI). Model 2 emerged as the most optimal, incorporating variables such as HER-2, ER, T-stage, Reg IV, and Treatment, among others. The area under the ROC curve (AUC) for Model 2 in the training dataset and test dataset was 0.837 (0.734–0.941) and 0.897 (0.775–1.00), respectively. Decision curve analysis (DCA) and clinical impact curve (CIC) further underscored the potential applications of the model in guiding clinical interventions for patients.

**Conclusion:**

The prediction of NACT pCR efficacy based on the expression of Reg IV in breast cancer tissue appears feasible; however, it requires further validation.

**Supplementary Information:**

The online version contains supplementary material available at 10.1007/s12282-024-01609-y.

## Instruction

The prevailing data on global cancer epidemiology underscores the status of breast cancer as the most prevalent malignancy afflicting the female population [1]. With advancements in treatment modalities, there has been a gradual decline in the recurrence rate of breast cancer [2]. However, a substantial proportion of patients, approximately 25–35%, experience disease progression to an advanced stage with limited therapeutic options available [3]. Neoadjuvant chemotherapy (NACT) has emerged as a standard and widely employed treatment regimen for breast cancer, demonstrating the potential to mitigate recurrence risk [4]. Notably, patients achieving pathological complete remission (pCR) through NACT exhibit a more favorable prognosis compared to those without complete pathological remission (nonpCR) [5]. However, existing literature indicates significant heterogeneity in the pCR response to NACT [6, 7]. Therefore, the exploration of specific biomarkers to predict NACT efficacy holds paramount significance. Regenerating islet-derived family member IV (Reg IV), a member of the calcium-dependent lectin gene superfamily, has been associated with tumor cell invasion and migration in various cancers, including colorectal, gastric, gallbladder, pancreatic, ovarian, prostate, and lung cancers, demonstrating a correlation with unfavorable prognosis and clinicopathological characteristics [8, 9]. Despite this, limited research has been conducted on the role of Reg IV in breast tumors. The present study endeavors to elucidate the expression of Reg IV protein in breast cancer and analyze its correlation with NACT efficacy, with the primary objective of establishing and validating a predictive model.

## Materials and methods

### Research participants

Data on formalin-fixed paraffin-embedded specimens from 104 patients diagnosed with NACT, who underwent puncture biopsy and received a diagnosis of invasive carcinoma, were collected at the Pathology Department of the First People’s Hospital of Lianyungang between September 2019 and December 2021. Inclusion criteria encompassed: (1) patients who had not undergone radiotherapy or tumor endocrine therapy prior to NACT; (2) patients with no detected distant metastatic lesions on imaging during the initial visit and without a history of other malignancies; (3) patients receiving a minimum of four courses of NACT; (4) patients undergoing surgery and pathological examination after their NACT; (5) patients possessing complete clinicopathologic data. Exclusion criteria involved: (1) patients with bilateral or secondary breast cancer, and those who were lactating or pregnant; (2) patients with distant metastases detected by imaging; (3) patients experiencing severe toxic reactions to chemotherapy leading to a chemotherapy delay exceeding 2 weeks or necessitating a switch to another regimen or treatment discontinuation for various reasons; (4) patients not undergoing surgical treatment post-chemotherapy; (5) patients with incomplete clinical data; and (6) patients unwilling to participate in the study. Approval for this study was granted by the Ethics Committee of the First People’s Hospital of Lianyungang (Approval No. KY-20230410002-01), and all participants duly signed the informed consent forms.

### Observation indicators

Patient characteristics and pathological features data were sourced from the medical record data system and pathological reporting system of our hospital, respectively. All specimens were stored in the specimen repository of the pathology department. The specimens underwent fixation in a 3.7% neutral formaldehyde solution, followed by routine processes of dehydration, transparency, waxing, and embedding. Subsequently, they were sliced at a thickness of 4 μm, subjected to staining using the immunohistochemical EnVision two-step method, and observed under a light microscope. The spare slices underwent baking in a 60 °C oven for 90 min, dewaxing with xylene, and hydration with gradient ethanol. The slices were subjected to blocking of endogenous peroxidase with 3% hydrogen peroxide. High-pressure repair (Reg IV) and EDTA repair (EGFR) were carried out using citrate buffer. The slices were then blocked with goat serum for 20 min, incubated with the primary antibody overnight at 4 °C (Reg IV, 1:100 dilution; EGFR, working solution), and subsequently incubated with the secondary antibody at 37 °C for 40 min. DAB was used for color development and hematoxylin for redyeing. PBS replaced the primary antibody to constitute the negative control group. Comprehensive scoring was conducted based on the product score of staining intensity and the percentage of positive cells. The following steps were employed: 5–10 high-power fields were randomly selected to calculate the average percentage of positive cells; scoring was based on the percentage of positive cells (0 points for no positive cells, 1 point for 1–25%, 2 points for 26–50%, 3 points for 51–75%, 4 points for 76–100%), and based on staining intensity (0 points for no staining, 1 point for light yellow, 2 points for claybank, 3 points for tan) (the immunohistochemical images of Reg IV protein are shown in Fig. S1). The score of the percentage of positive cells was multiplied by the score of staining intensity. A total score equal to or less than 3 was deemed negative, while a score exceeding 3 was considered positive. The evaluation of the results was conducted by two pathologists with senior professional titles in a double-blind manner.

Breast cancer staging was conducted following the tumor–node–metastasis (TNM) system, established by the International Union Against Cancer (UICC) [10]. In accordance with this system, tumors with a maximum diameter of less than or equal to 20 mm are categorized as T1, those measuring between 20 and 50 mm as T2, and those exceeding 50 mm as T3. Lymph node involvement is classified as N0 in the absence of metastasis, N1 for one to three metastatic lymph nodes, N2 for four to eight metastatic lymph nodes, and N3 for nine or more metastatic lymph nodes. Estrogen receptor (ER) and progesterone receptor (PR) expression in tumor cells equal to or greater than 1% is considered positive expression, with 1–30% classified as low expression and > 30% as high expression. Conversely, expression levels less than 1% are regarded as negative expression. Determination of HER-2 positivity or negativity was carried out in accordance with the guidelines from the American Society of Clinical Oncology (ASCO) and the College of American Pathologists (CAP) for HER-2 testing in breast cancer [11]. Positive HER-2 expression is defined by immunohistochemical (IHC) staining of 3 + (indicating strong expression in more than 30% of invasive tumor cells) and fluorescence in situ hybridization (FISH) results demonstrating more than six copies of the HER-2 gene per cell nucleus or a FISH ratio (chromosome 17/HER-2 gene signal) exceeding 2.2. Negative HER-2 expression is identified by IHC staining of 0 or 1 + , with FISH results displaying less than 4.0 HER-2 gene copies per nucleus or a FISH ratio below 1.8. HER-2 (3 +), as well as HER-2 (2 +) with FISH ( +),collectively categorized as HER-2 positive, while HER-2 (0), (1 +), or (2 +) with concurrent FISH (−) all defined as HER-2 negative. Ki-67 expression less than 20% is considered low expression, whereas equal to or greater than 20% is classified as high expression.

### Efficacy assessment

In accordance with the eighth edition of the American Joint Committee on Cancer (AJCC), pCR is defined as the absence of invasive breast cancer (ypT0/ypTis, ypN0) in the primary site/axillary lymph nodes of surgical specimens following NACT. This definition does not impose limitations on the presence of residual non-invasive breast cancer, such as ductal carcinoma in situ.

### Statistical methods

Statistical analyses and visualization were conducted using R 4.0.2 software. Clinical characteristics between groups were compared using the Student’s *t* test or Wilcoxon rank-sum test for continuous variables, and the Chi-squared test or Fisher’s exact test (as appropriate) for categorical variables. Variable screening was carried out using logistic regression, random forest, and eXtreme Gradient Boosting (Xgboost) methods. The models were assessed using the area under the ROC curve (AUC) and calibration curve. NRI and IDI were used to assess differences between various models [12, 13]. Nomograms were established to visualize the models. Decision curve analysis (DCA) and clinical impact curve (CIC) were employed to assess the clinical benefits of the nomogram. Statistical significance was considered at a *p* value less than 0.05.

## Result

### Research queue

In this study, we enrolled a total of 104 patients, among whom positive Reg IV expression was observed in breast cancer tissues in 60 patients (57.5%). The rate of Reg IV expression varied across different types of breast cancer, with rates of 61.3% (13/21) in triple-negative breast cancer (TNBC), 45.8% (22/48) in luminal type, and 71.4% (25/35) in HER-2 positive type. Among Reg IV-positive patients, approximately 48.3% (29/60) achieved a pCR in NACT, whereas the likelihood of Reg IV-negative patients achieving pCR was around 20% (8/40). The patients were stratified into training and validation datasets in a randomized manner, following a 7:3 ratio (refer to Table S1 for specific details). Subsequently, the patients were categorized based on whether they received NACT treatment, and individual indicators were analyzed between groups within different datasets. The results indicated that the overall characteristics of the data were comparable across the various datasets (Table 1). We calculated the positive expression of Reg IV protein in each type and found that a total of 21 patients were included in TNBC, of whom 13 were Reg IV positive, accounting for 61.9%; among 35 patients with luminal, 25 were Reg IV positive, accounting for 71.4%. Among the HER-2 positive patients, 22 were Reg IV positive, accounting for 45.8%. Data analysis showed that there were differences in the expression of Reg IV in different subtypes, especially in luminal and HER-2 positive types.

**Table 1:** Baseline by PCR

	All dataset				Training dataset				Test dataset			
Variable	Overall, *N* = 104^a^	Non PCR, *N* = 67^a^	PCR, *N* = 37^a^	*p* Value^b^	Overall, * N* = 71^a^	Non PCR, *N* = 46^a^	PCR, *N* = 25^a^	*p* Value^b^	Overall, * N* = 33^a^	Non PCR, *N* = 21^a^	PCR, *N* = 12^a^	*p* Value^b^
Treatment, *n *(%)				< 0.001				0.028				0.007
AC	3 (2.9%)	3 (4.5%)	0 (0%)		3 (4.2%)	3 (6.5%)	0 (0%)		0 (0%)	0 (0%)	0 (0%)	
AC-T	16 (15%)	15 (22%)	1 (2.7%)		8 (11%)	7 (15%)	1 (4.0%)		8 (24%)	8 (38%)	0 (0%)	
AC-THP	5 (4.8%)	1 (1.5%)	4 (11%)		5 (7.0%)	1 (2.2%)	4 (16%)		0 (0%)	0 (0%)	0 (0%)	
TAC	25 (24%)	20 (30%)	5 (14%)		16 (23%)	13 (28%)	3 (12%)		9 (27%)	7 (33%)	2 (17%)	
TCbH	2 (1.9%)	2 (3.0%)	0 (0%)		1 (1.4%)	1 (2.2%)	0 (0%)		1 (3.0%)	1 (4.8%)	0 (0%)	
TCbHP	37 (36%)	17 (25%)	20 (54%)		26 (37%)	13 (28%)	13 (52%)		11 (33%)	4 (19%)	7 (58%)	
TCHP	5 (4.8%)	1 (1.5%)	4 (11%)		3 (4.2%)	1 (2.2%)	2 (8.0%)		2 (6.1%)	0 (0%)	2 (17%)	
THP	7 (6.7%)	4 (6.0%)	3 (8.1%)		5 (7.0%)	3 (6.5%)	2 (8.0%)		2 (6.1%)	1 (4.8%)	1 (8.3%)	
TP	4 (3.8%)	4 (6.0%)	0 (0%)		4 (5.6%)	4 (8.7%)	0 (0%)		0 (0%)	0 (0%)	0 (0%)	
Age, *n*(%)				0.2				0.3				0.6
< 40	16 (15%)	8 (12%)	8 (22%)		12 (17%)	6 (13%)	6 (24%)		4 (12%)	2 (9.5%)	2 (17%)	
≥ 40	88 (85%)	59 (88%)	29 (78%)		59 (83%)	40 (87%)	19 (76%)		29 (88%)	19 (90%)	10 (83%)	
Type, *n*(%)				< 0.001				< 0.001				0.2
TNBC	21 (20%)	17 (25%)	4 (11%)		13 (18%)	11 (24%)	2 (8.0%)		8 (24%)	6 (29%)	2 (17%)	
LUMI	35 (34%)	12 (18%)	23 (62%)		25 (35%)	8 (17%)	17 (68%)		10 (30%)	4 (19%)	6 (50%)	
HER2	48 (46%)	38 (57%)	10 (27%)		33 (46%)	27 (59%)	6 (24%)		15 (45%)	11 (52%)	4 (33%)	
Clinic stage, *n *(%)				0.6				0.6				> 0.9
T2	40 (38%)	27 (40%)	13 (35%)		26 (37%)	18 (39%)	8 (32%)		14 (42%)	9 (43%)	5 (42%)	
T3	64 (62%)	40 (60%)	24 (65%)		45 (63%)	28 (61%)	17 (68%)		19 (58%)	12 (57%)	7 (58%)	
Menstrual status, *n*(%)				0.6				> 0.9				0.5
Premenopause	41 (39%)	25 (37%)	16 (43%)		28 (39%)	18 (39%)	10 (40%)		13 (39%)	7 (33%)	6 (50%)	
Postmenopause	63 (61%)	42 (63%)	21 (57%)		43 (61%)	28 (61%)	15 (60%)		20 (61%)	14 (67%)	6 (50%)	
T-stage, *n*(%)				0.7				0.4				0.5
T2	79 (76%)	50 (75%)	29 (78%)		57 (80%)	35 (76%)	22 (88%)		22 (67%)	15 (71%)	7 (58%)	
T3	25 (24%)	17 (25%)	8 (22%)		14 (20%)	11 (24%)	3 (12%)		11 (33%)	6 (29%)	5 (42%)	
N-stage, *n*(%)				0.8				0.3				0.3
Neg	38 (37%)	24 (36%)	14 (38%)		28 (39%)	16 (35%)	12 (48%)		10 (30%)	8 (38%)	2 (17%)	
Pos	66 (63%)	43 (64%)	23 (62%)		43 (61%)	30 (65%)	13 (52%)		23 (70%)	13 (62%)	10 (83%)	
ER, *n*(%)				< 0.001				0.005				0.064
Neg	50 (48%)	24 (36%)	26 (70%)		35 (49%)	17 (37%)	18 (72%)		15 (45%)	7 (33%)	8 (67%)	
Pos	54 (52%)	43 (64%)	11 (30%)		36 (51%)	29 (63%)	7 (28%)		18 (55%)	14 (67%)	4 (33%)	
PR, *n*(%)				0.008				0.011				0.5
Neg	70 (67%)	39 (58%)	31 (84%)		49 (69%)	27 (59%)	22 (88%)		21 (64%)	12 (57%)	9 (75%)	
Pos	34 (33%)	28 (42%)	6 (16%)		22 (31%)	19 (41%)	3 (12%)		12 (36%)	9 (43%)	3 (25%)	
HER-2, *n*(%)				< 0.001				< 0.001				0.002
Neg	47 (45%)	41 (61%)	6 (16%)		30 (42%)	26 (57%)	4 (16%)		17 (52%)	15 (71%)	2 (17%)	
Pos	57 (55%)	26 (39%)	31 (84%)		41 (58%)	20 (43%)	21 (84%)		16 (48%)	6 (29%)	10 (83%)	
Ki-67, *n*(%)				0.6				0.7				0.13
< 20	23 (22%)	16 (24%)	7 (19%)		18 (25%)	11 (24%)	7 (28%)		5 (15%)	5 (24%)	0 (0%)	
≥ 20	81 (78%)	51 (76%)	30 (81%)		53 (75%)	35 (76%)	18 (72%)		28 (85%)	16 (76%)	12 (100%)	
Reg IV, *n*(%)				0.002				0.022				0.024
Neg	44 (42%)	36 (54%)	8 (22%)		30 (42%)	24 (52%)	6 (24%)		14 (42%)	12 (57%)	2 (17%)	
Pos	60 (58%)	31 (46%)	29 (78%)		41 (58%)	22 (48%)	19 (76%)		19 (58%)	9 (43%)	10 (83%)	

^a^Mean (SD) or median (IQR) or frequency (%)

^b^Fisher’s exact test; Pearson’s Chi-squared test

### Analysis of correlation and covariance between variables

**Fig. 1:**
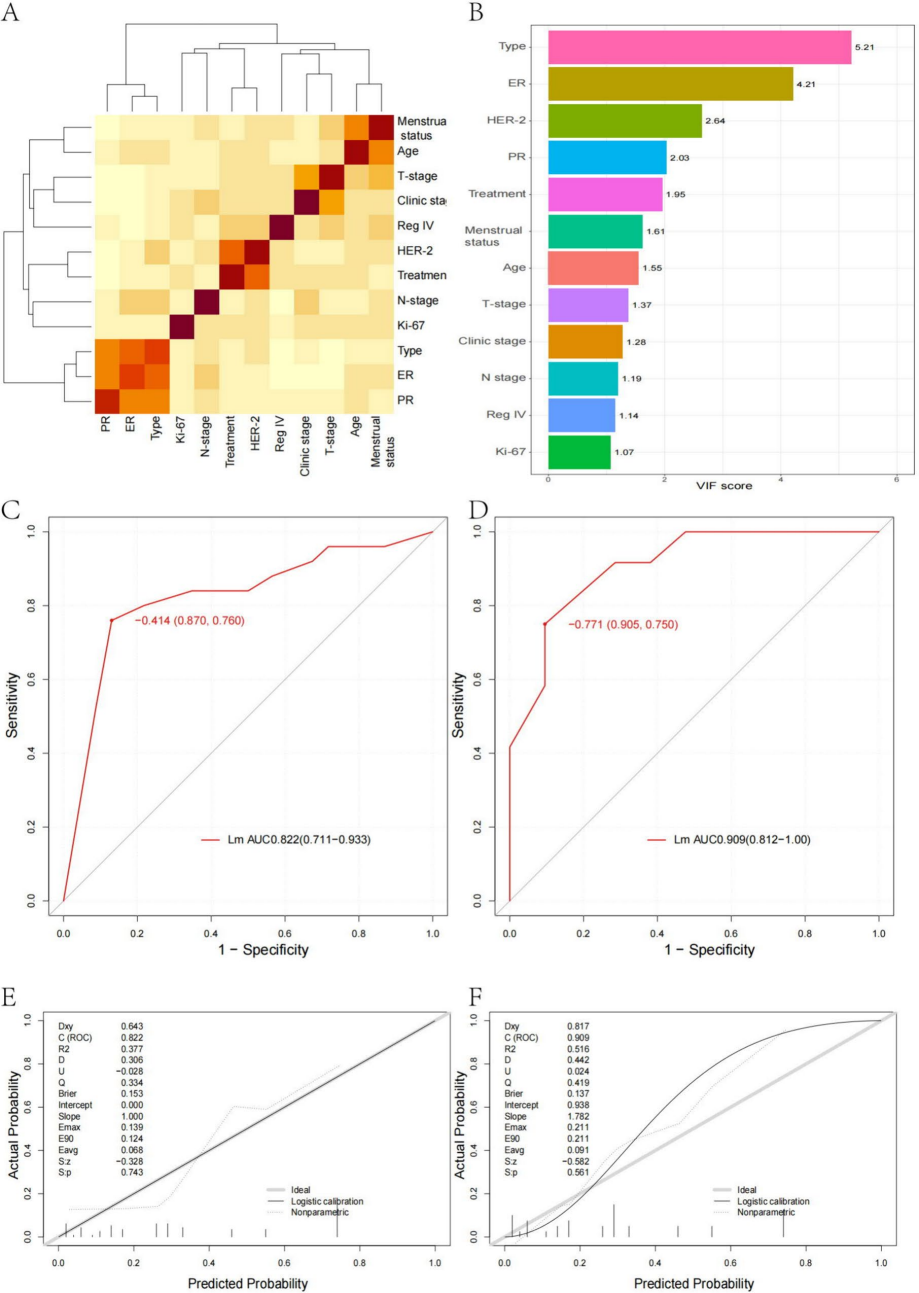
**A** Significant clustering is observed in ER, PR, and the type, particularly between Treatment and HER-2, Age and Menstrual status, as well as T_stage and Clinic_stage. **B** The VIFs between variables indicate that overall collinearity is fair. However, the VIF of Type slightly exceeds 5, and the VIF of ER approaches 5. **C** The AUC in the training dataset for the model based on linear logistic regression is 0.822 (0.711–0.933). **D** The AUC in the training dataset for the model based on logistic regression is 0.909 (0.812–1.000). **E**, **F** In the training dataset, the new model exhibits a Dxy value of 0.643, an *R*^2^ value of 0.377, a Brier value of 0.153, and a C-index value of 0.822. In the test dataset, these values are Dxy 0.817, *R*^2^ 0.516, Brier 0.137, and C-index 0.909, respectively

Cluster analysis revealed significant clustering in ER, PR, and type, as well as in Treatment and HER-2, Age and Menstrual status, and T-stage and Clinical stage. This indicates the presence of high-dimensional data. Subsequently, an exploration of the variance inflation factor (VIF) between the individual variables was conducted, indicating a generally fair level of covariance. However, it was noted that the VIF of the type exceeded 5, and the VIF of ER was close to 5, indicating the need for further screening of the included variables (refer to Fig. 1A, B). We further explored the correlation between pCR and Reg IV, and the results were rho = 0.311, *p* value = 0.001304, indicating a statistically significant correlation between the two.

### Modeling

The model Lm was established through univariate logistic regression analysis and the AUC. Variables with *p* < 0.05 and an AUC greater than 0.6 were included for multivariate analysis (Table 2) (Figure S2). The AUC for model Lm in the training dataset was 0.822 (0.711–0.933), and in the test dataset, it was 0.909 (0.812–1.00). The calibration curve demonstrated that model Lm exhibited good predictive performance and stability (Fig. 1C, D), with a Dxy value of 0.643, an *R*^2^ value of 0.377, a Brier value of 0.153, and a C-index value of 0.822 in the training dataset, and a Dxy value of 0.817, an *R*^2^ value of 0.516, a Brier value of 0.137, and a C-index value of 0.909 in the test dataset (Fig. 1E, F).

**Table 2:** Univariate and multivariate analysis and ROC analysis

Characteristic	Univariate			ROC		Multivariate		
	OR^a^	95% CI^a^	*p* Value	AUC	95% CI^a^	OR^a^	95% CI^a^	*p* Value
Treatment	1.14	0.89, 1.44	0.30	0.58	0.45, 0.71			
Age	0.47	0.14, 1.67	0.26	0.56	0.46, 0.65			
Type	0.72	0.38, 1.37	0.32	0.60	0.47, 0.73			
Clinic stage	1.36	0.49, 3.82	0.55	0.54	0.42, 0.65			
Menstrual status	0.96	0.36, 2.61	0.94	0.50	0.38, 0.63			
T-stage	0.43	0.11, 1.73	0.24	0.56	0.47, 0.65			
N-stage	0.57	0.21, 1.56	0.28	0.57	0.44, 0.69			
ER	0.23	0.08, 0.66	0.006	0.68	0.56, 0.79	0.08	0.01, 0.60	0.014
PR	0.19	0.05, 0.74	0.17	0.65	0.55, 0.74	0.58	0.09, 3.22	0.5
HER-2	6.82	2.02, 23.07	0.002	0.70	0.60, 0.81	6.98	2.00, 30.0	0.004
Ki-67	0.81	0.27, 2.44	0.71	0.52	0.41, 0.63			
Reg IV	3.45	1.17, 10.22	0.025	0.64	0.53, 0.75	3.71	1.24, 11.04	0.019

^a^*OR* odds ratio; *CI* confidence interval

**Fig. 2:**
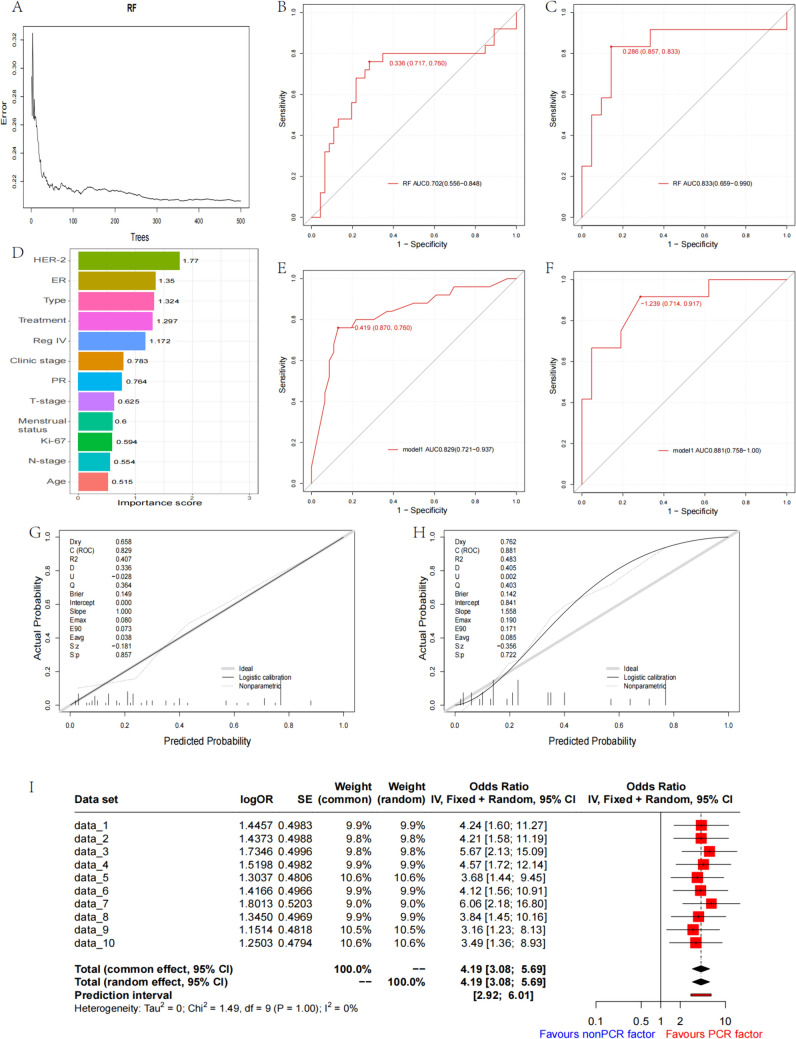
**A** The random forest error consistently decreases with an increasing number of trees. **B**, **C** The random forest model exhibits an AUC of 0.702 (0.556–0.848) and 0.833 (0.659–0.990) in the training and test dataset. **D** The variable importance ordering in random forests. **E**, **F** The AUC for model 1 is 0.829 (0.721–0.937) in the training dataset and 0.881 (0.758–1.00) in the test dataset. **G**, **H** The calibration curve for model 1 indicates that in the training dataset, the Dxy value was 0.658, the *R*^2^ value 0.407, the Brier value 0.149, and the C-index value 0.829, while in the test dataset, the Dxy was 0.762, the *R*^2^ 0.483, the Brier 0.142, and the C-index 0.881. **I** Meta-integration of tenfold resampling data through random-effect model and fixed-effect model suggested that Reg IV protein expression was beneficial to PCR for neoadjuvant chemotherapy

The RF model was trained in the training dataset using the random forest method, resulting in an AUC of 0.702 (0.556–0.848) in the training dataset and 0.833 (0.659–0.990) in the test dataset (Fig. 2B, C). The prediction performance of the random forest method was slightly lower than that of the logistic regression method in the training dataset, and similar in the test dataset. We calculated the weights of the variables in the random forest model, and the top five variables were included to build model 1, which consisted of HER-2, ER, Type, Treatment, and Reg IV (Fig. 2D). The AUC for model 1 in the training dataset was 0.829 (0.721–0.937), and in the test dataset, it was 0.881 (0.758–1.00) (Fig. 2E, F). The calibration curve for model 1 revealed that in the training dataset, the Dxy value was 0.658, the *R*^2^ value 0.407, the Brier value 0.149, and the C-index value 0.829 (Fig. 2G), while in the test dataset, the Dxy was 0.762, the *R*^2^ 0.483, the Brier 0.142, and the C-index 0.881 (Fig. 2H). We further explored the imbalance in the data. We randomly sampled the data with the method of tenfold sampling, and analyzed the relationship between Reg IV protein and PCR for each resampled sample. Finally, fixed- or random-effect models were used to combine the data. However, whether in a single sample of data or after the combination, Reg IV protein positive was beneficial to obtain PCR for neoadjuvant chemotherapy (Fig. 2I).

**Fig. 3:**
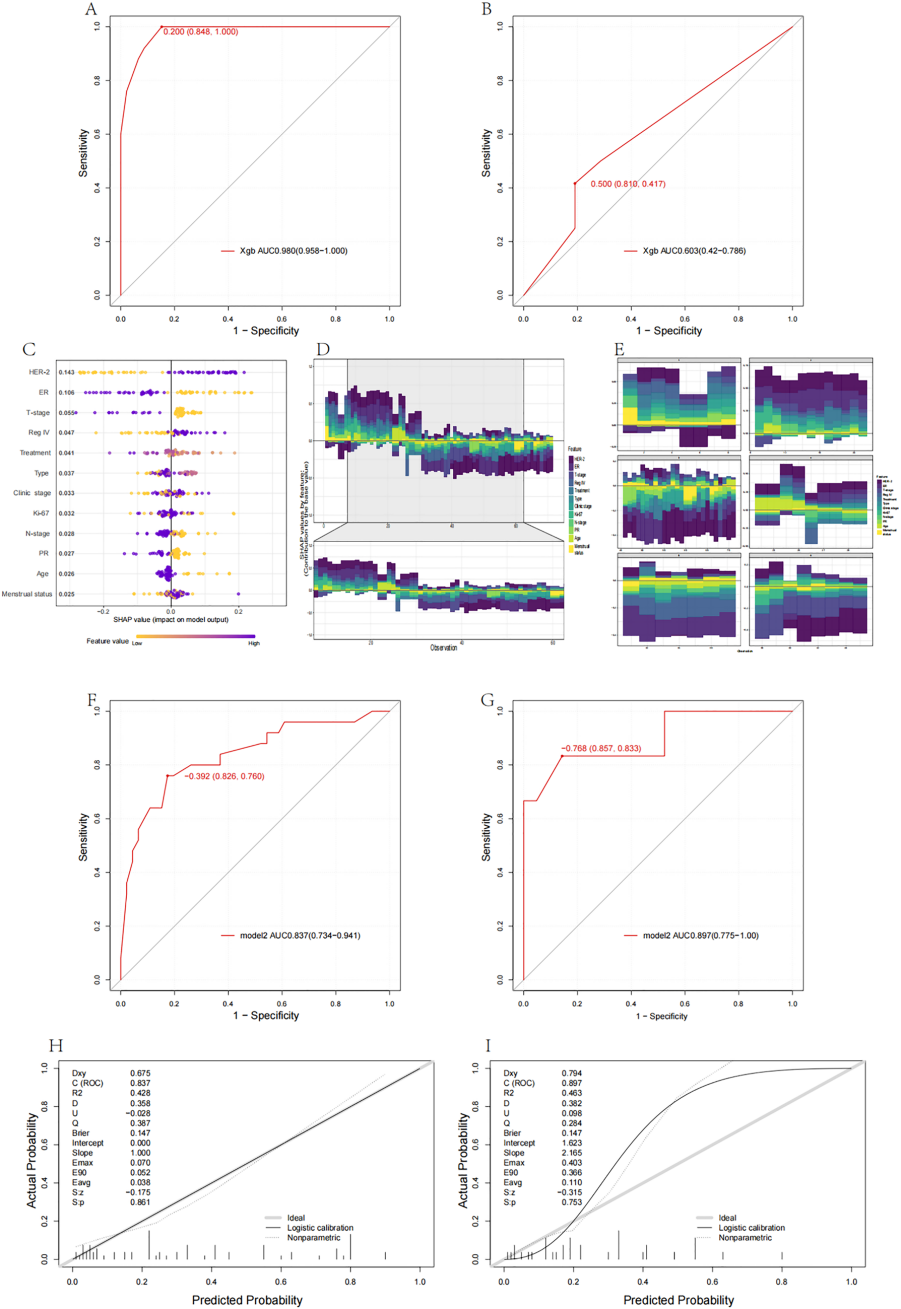
**A**, **B** The AUC for the training model based on Xgboost in the training dataset is 0.980 (0.958–1.000), and the AUC for the validation model in the test dataset is 0.603 (0.042–0.786). **C**–**E** The model was interpreted using SHAP to assess the importance of variables, and the top five variables included in model 2 are HER-2, ER, T_Stage, Reg IV, and Treatment. **F**, **G** Model 2 exhibits an AUC of 0.837 (0.734–0.941) in the training dataset and 0.897 (0.775–1.00) in the test dataset. **H**, **I** The calibration curve for model 2 indicates a Dxy value of 0.675, an *R*^2^ value of 0.428, a Brier value of 0.147, and a C-index value of 0.837 in the training dataset. In the test dataset, the Dxy value is 0.920, the *R*.^2^ value is 0.770, the Brier value is 0.072, and the C-index is 0.897

The Xgboost method was used to train the model, resulting in an AUC of 0.980 (0.958–1.000) in the training dataset (Fig. 3A). However, the performance of the model in the test dataset was suboptimal, with an AUC of 0.603 (0.042–0.786) (Fig. 3B). Given this observed inadequacy in the validation set, the model was interpreted using SHAP to assess the importance of variables, and the top five variables in terms of importance were selected to establish model 2. These variables included HER-2, ER, T_Stage, Reg IV, and Treatment (Fig. 3C–E). The AUC for model 2 was 0.837 (0.734–0.941) in the training dataset and 0.897 (0.775–1.00) in the test dataset (Fig. 3F, G). The calibration curve for model 2 indicated a Dxy value of 0.675, an *R*^2^ value of 0.428, a Brier value of 0.147, and a C-index value of 0.837 in the training dataset (Fig. 3H). In the test dataset, the calibration curve revealed a Dxy value of 0.794, an *R*^2^ value of 0.463, a Brier value of 0.147, and a C-index value of 0.897. (Fig. 3I).

### Comparison of models and their validation, and display of nomograms, DCA, and CIC curves

**Fig. 4:**
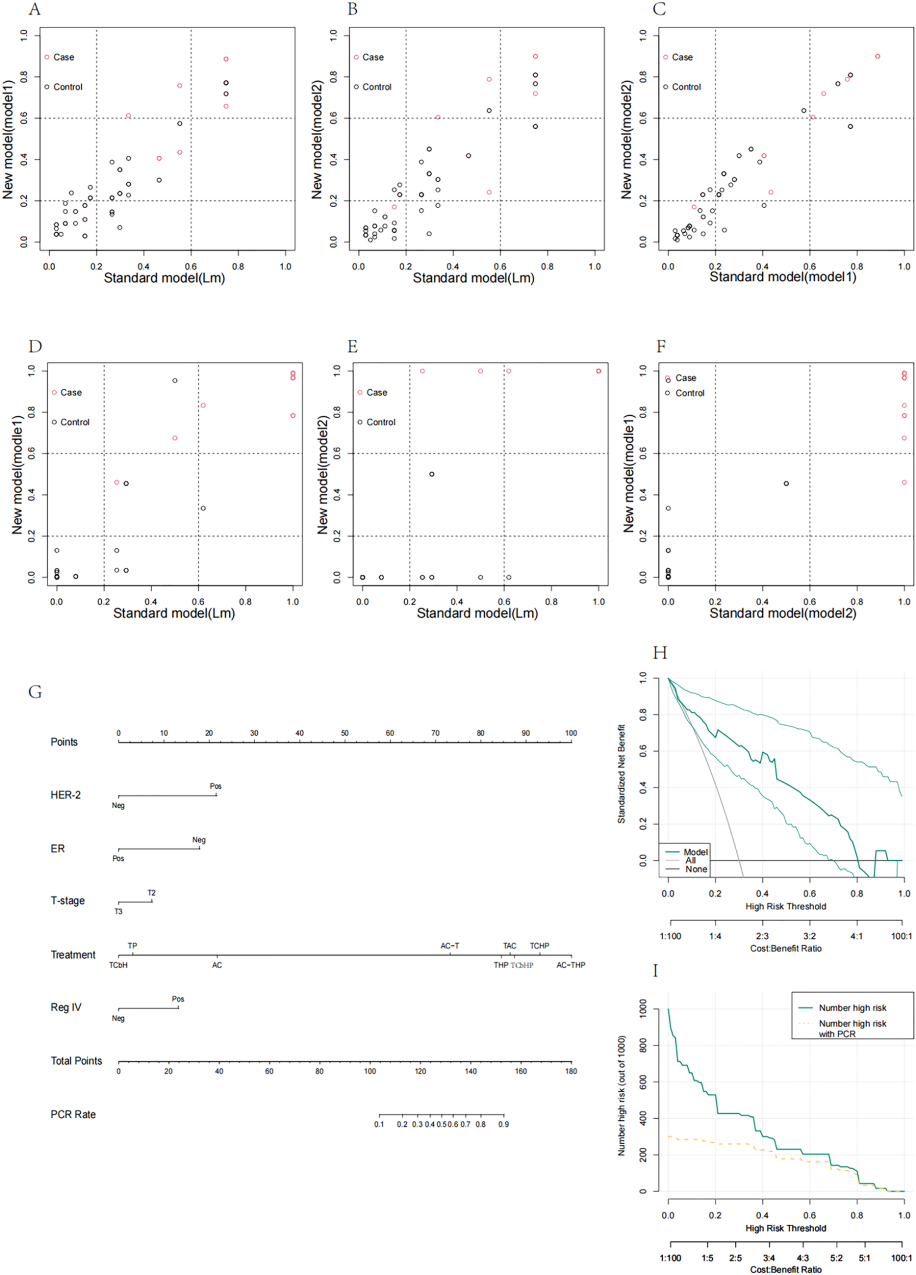
**A**–**C** represent the pairwise comparisons of model 1, model 2, and model Lm in the training dataset, indicating no significant differences. **D**–**F** are pairwise comparisons of model 1, model 2, and model Lm in the test dataset. Model 2 is superior to model Lm, and there is no significant difference between model 1 and model 2. **G** presents nomogram based on model 2 in the full dataset, while **H** and **I** are the DCA and CIC curves of model 2, respectively

The three models, including the logistic regression-based model (Lm), were established, and while there was no significant difference in the AUC, the included variables were not entirely consistent. Further comparisons between the three models were conducted using NRI and IDI in both the training and validation sets. In the training set, comparing Lm with model 1 yielded NRI: 0.08 [−0.069–0.229], *p* value: 0.29266; IDI: 0.0255 [−0.0187–0.0697], *p* value: 0.25861, indicating no statistically significant difference between the two models (Fig. 4A). Similarly, comparing Lm with model 2 resulted in NRI: −0.0252 [−0.275–0.2246], *p* value: 0.84315; IDI: 0.0356 [−0.0193–0.0906], *p* value: 0.20367, with no statistical difference (Fig. 4B). When comparing model 1 with model 2, the results were NRI: −0.1052 [−0.3208–0.1104], *p* value: 0.33875; IDI: 0.0102 [−0.0308–0.0512], *p* value: 0.62684, indicating that model 1 was slightly inferior to model 2, but with no statistically significant difference (Fig. 4C). In the validation set, comparing Lm with model 1 resulted in NRI 0.2381 [−0.0849–0.5611], *p* value: 0.14851; IDI: 0.0477 [−0.0649–0.1603], *p* value: 0.40603, indicating no statistically significant difference (Fig. 4D). The comparison between Lm and model 2 revealed NRI: 0.3333 [0.0078–0.6589], *p* value: 0.04477; IDI: 0.2671 [0.1004–0.4338], *p* value: 0.00168, indicating that model 2 was superior to Lm (Fig. 4E). Additionally, the comparison between model 1 and model 2 indicated NRI: −0.1786 [−0.3791–0.022], *p* value: 0.08094; IDI: −0.2194 [−0.3498 to −0.089], *p* value: 0.00098, signifying that model 2 outperformed model 1 (Fig. 4F). Based on the analysis, model 2 was selected as the best-performing model and was visualized. We created a nomogram based on model 2 in the full dataset (Fig. 4G). The final DCA and CIC curves reflected the excellent performance of the model in clinical applications (Fig. 4H, I).

## Discussion

The use of NACT is progressively on the rise, and accurately forecasting the effectiveness of NACT with various regimens holds paramount significance. Beyond conventional predictors such as clinical staging and lymph node metastasis, ongoing efforts are directed toward enhancing predictive accuracy [14, 15]. Elevated expression of Reg IV has been implicated in promoting tumor cell invasion and migration [16, 17]. Furthermore, Reg IV has the capability to activate EGFR, thereby expediting cell proliferation while impeding apoptosis. Despite the well-established role of Reg IV in these processes, there are a few studies investigating its potential as a predictor of neoadjuvant efficacy in breast cancer. In this study, we explored the predictive efficacy of Reg IV within the context of NACT for breast cancer.

This study encompassed a cohort of 104 patients, among whom positive Reg IV expression was identified in breast cancer tissues for 60 patients, constituting 57.5% of the total. The prevalence of Reg IV expression exhibited variability among different breast cancer types, with rates of 61.3% (13/21) in TNBC, 45.8% (22/48) in luminal type, and 71.4% (25/35) in HER-2 positive type. Within the subset of patients with positive Reg IV, approximately 48.3% (29/60) achieved pCR in NACT, in contrast to patients with negative Reg IV, where the likelihood of achieving pCR was approximately 20% (8/40). These findings indicate a potential predictive role of Reg IV in determining the efficacy of NACT. Interestingly, however, the pCR rates of patients with subtypes of TNBC, luminal, and Her2 were 11%, 62%, and 27%, respectively, which seems to be different compared to conventional reported pCR rates after NACT. We found that the expression of Reg IV protein was not identical in different subtypes of breast cancer, with 61.9% and 71.4% of patients with TNBC breast cancer and luminal breast cancer being positive for Reg IV protein. However, in HER-2 positive patients, the proportion of Reg IV protein-positive patients was only 45.8%. We speculated that the reasons may be: first, it is true that the expression of Reg IV is inconsistent in different breast subtypes, but this solution needs to be further expanded to confirm; second, there is a certain distribution bias due to the small amount of data of included patients. The training dataset and the test dataset were randomly assigned, but there seemed to be bias between the two datasets in terms of tumor molecular type, clinical stage, and Ki67, which may be caused by the small sample size. To further explore the problem of imbalance in the data cohort, we adopted the method of 10% sampling to randomly sample the data. The relationship between Reg IV protein and PCR was analyzed for each resampled sample, and the fixed- or random-effect model was used to combine the samples. We found that random sampling did show uneven data, but the relationship between Reg IV protein and PCR was still well reflected after the overall combination. Therefore, we speculate that the differences between some variables of the training set and the verification set may be caused by the small sample size, and the sample size needs to be further increased.

We employed three methods—linear regression, random forest, and gradient boosting. Linear regression model is simple in structure and easy to understand and implement. The calculation process of linear regression is relatively simple, and it can fit a large amount of data in a short time. For many practical problems, linear regression model can provide relatively accurate prediction results, but it is not enough to fit nonlinear relations and is more sensitive to outliers, so it needs to consider collinearity, which makes its use have certain limitations. The essence of a random forest is a collection of decision trees, and each decision tree has a slightly different strategy. The idea of a random forest is that each tree may have a relatively good prediction, but there may be an overfitting of some data. In addition, if the prediction of each tree is good, each tree may have overfitting in different ways, so overfitting can be reduced by means of average value, which can not only reduce overfitting, but also maintain good prediction ability, and at the same time, the importance of variables can be ranked. Unlike random forests, Xgboost constructs decision trees in a continuous manner, with each tree trying to modify the errors of the previous tree. The main idea is to combine multiple simple models and iteratively improve performance, which can achieve high accuracy. Here, we choose three commonly used modeling methods. The main purpose is to explore simple and easy methods, such as whether linear regression has good predictive efficiency, and also take into account classifier integration, including random forest and gradient lifting, and the difference between the two is that random forest is a parallel tree and gradient lifting is a progressive tree. We compared the differences among the three models and discovered that the linear regression model exhibited superior and more stable performance in both the training and validation sets. The predictive efficacy of the random forest model in the validation set is observed to be inferior to that in the training set, and there exists a notable inadequacy in its stability. In contrast, while the gradient boosting model demonstrated exceptionally high predictive efficacy in the training set, its performance markedly declined in the validation set, indicating limitations in generalization ability. This discrepancy may stem from the inherent requirements of large normative datasets for effective learning in supervised learning methods, a constraint exacerbated by the relatively small number of patient cases in our study. Further scrutiny involved variable screening, and the top five variables were extracted from both random forest and Xgboost models, including HER-2, ER, Type, Treatment, and Reg IV. Subsequently, a new model was established and compared with the variables selected by the linear model (Lm). The AUC for the Lm model in the training dataset was 0.822 (0.711–0.933), and in the test dataset, it was 0.909 (0.812–1.00), demonstrating robust predictive performance and stability. The calibration curve corroborated these findings, indicating favorable predictive performance in both datasets. Comparatively, model 1, incorporating variables from the linear model, exhibited an AUC of 0.829 (0.721–0.937) in the training dataset and 0.881 (0.758–1.00) in the test dataset. The calibration curve for model 1 demonstrated sound predictive performance in both datasets. Model 2, featuring the top five variables from the Xgboost model, displayed an AUC of 0.837 (0.734–0.941) in the training dataset and 0.897 (0.775–1.00) in the test dataset. The calibration curve for model 2 revealed a Dxy value of 0.675, an *R*^2^ value of 0.428, a Brier value of 0.147, and a C-index value of 0.837 in the training dataset. In the test dataset, model 2 displayed a Dxy value of 0.920, an *R*^2^ value of 0.77, and a Brier value of 0.072. Comparisons of models with different variables within the linear regression framework revealed similar predictive efficacy. Further assessment through NRI and IDI led to the identification of model 2 as the optimal choice, featuring five key variables: HER-2, ER, T_stage, Reg IV, and Treatment. This analysis underscored the significance of HER-2 expression, HR status, tumor size, Reg IV expression, and treatment regimen as pivotal indicators for determining the achievement of pCR following NACT. These findings align with current clinical practices, where targeted therapies in patients with positive HER-2 significantly enhance pCR rates [18], patients with positive ER receiving NACT exhibit lower pCR rates [19], and neoadjuvant endocrine therapies targeting HR positivity are relevant [20]. However, larger tumor sizes are associated with relatively lower pCR rates, and the efficacy of different neoadjuvant regimens varies. The inclusion of Reg IV expression as an indicator further enhanced the predictive efficacy and generalization ability of the model. DCA and CIC curves provided additional support, demonstrating the potential for clinical benefits when using this model for benefit assessment. The CIC curves specifically highlighted the strong discriminatory power of the model, reinforcing its utility in clinical decision-making processes related to NACT.


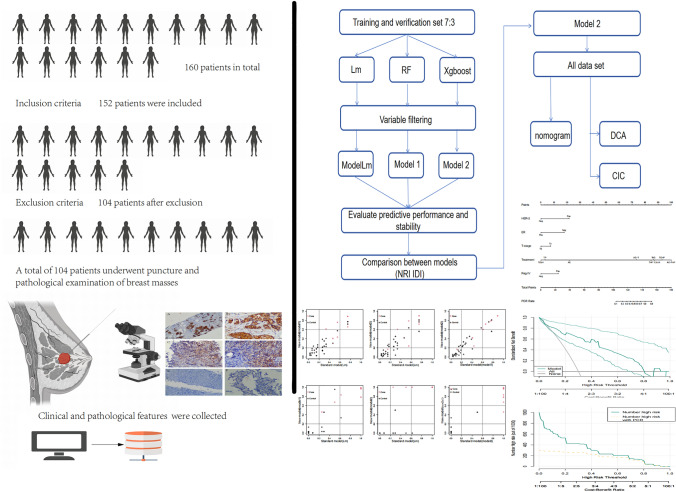


There are some limitations in this study. Primarily, the data analyzed is confined to specific time frames and medical institutions, potentially restricting its generalizability to broader populations. Additionally, the model construction necessitates further external validation to ensure its robustness and reliability. The inherent complexities in clinical practice pose challenges to the practical application of the model.

## Conclusion

In conclusion, this study has advanced the understanding of predicting the pCR rate in patients diagnosed with breast cancer undergoing NACT by leveraging Reg IV. The establishment and comparison of multiple models have enabled the identification of key factors, resulting in the development of a predictive model that is not only effective but also interpretable. However, the practical application of the model requires further validation in broader clinical settings to ensure its reliability and generalizability.

### Supplementary Information

Below is the link to the electronic supplementary material.

Figure S1 The immunohistochemical images of Reg IV protein. A: Reg IV protein immunohistochemical positive (×40). B: Reg IV protein immunohistochemistry positive (×200). C: Reg IV protein immunohistochemical weak positive (×40). D: Reg IV protein immunohistochemical weak positive (×200). E: Reg IV protein immunohistochemical negative (×40). F: Reg IV protein immunohistochemical negative (×200). Supplementary file1 (TIF 15097 KB)

Figure S2 ROC curves for each variable in univariate analysis for predicting pCR. Supplementary file2 (TIF 15112 KB)

Supplementary file3 (DOCX 19 KB)

## Data Availability

The datasets used and/or analyzed during the current study are available from the corresponding author upon reasonable request.

## Abbreviations

Reg IV: Regenerating islet-derived family member IV
PCR: Pathologic complete response
TNM: Tumor–node–metastasis
FISH: Fluorescence in situ hybridization
UICC: Union for International Cancer Control
AJCC: American Joint Committee on Cancer
NACT: Neoadjuvant chemotherapy
AUC: Area under the curve
NRI: Net reclassification improvement
IDI: Integrated discrimination improvement
DCA: Decision curve analysis
CIC: Clinical impact curve
Xgboost: EXtreme Gradient Boosting
ROC: Receiver operating characteristic curve
SHAP: Shapley Additive exPlanation
VIF: Variance inflation factor
