# Supplementary figures and images for: Development and validation of a neoadjuvant chemotherapy pathological complete remission model based on Reg IV expression in breast cancer tissues: a clinical retrospective study
Source: Breast Cancer. 2024 Jul 8;31(5):955–68. doi: 10.1007/s12282-024-01609-y (PMC11341653; doi:10.1007/s12282-024-01609-y)

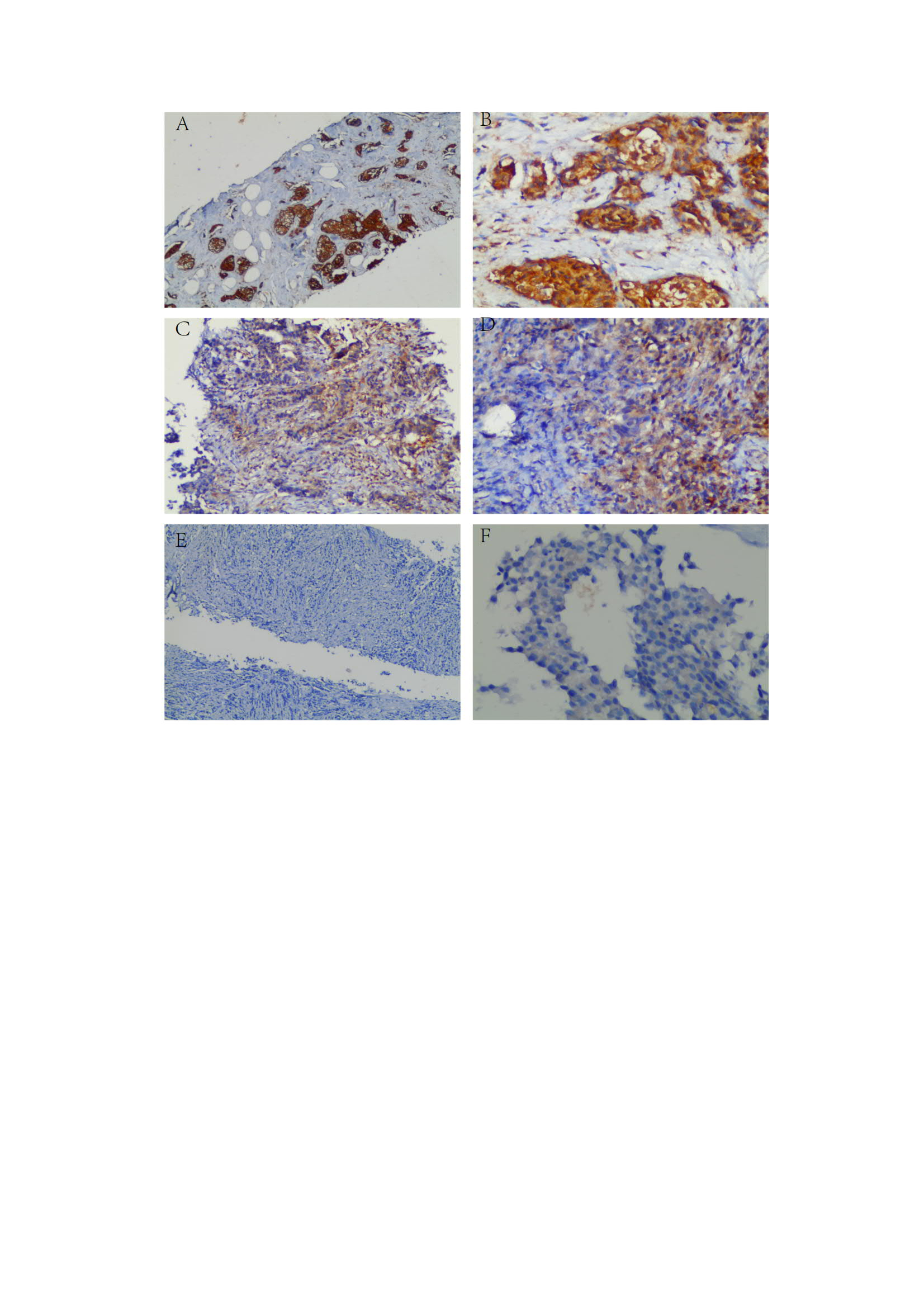

Supplement: Supplementary file 1 — The immunohistochemical images of Reg IV protein. A: Reg IV protein immunohistochemical positive (×40). B: Reg IV protein immunohistochemistry positive (×200). C: Reg IV protein immunohistochemical weak positive (×40). D: Reg IV protein immunohistochemical weak positive (×200). E: Reg IV protein immunohistochemical negative (×40). F: Reg IV protein immunohistochemical negative (×200). Supplementary file1 (TIF 15097 KB) [file 12282_2024_1609_MOESM1_ESM.tif]

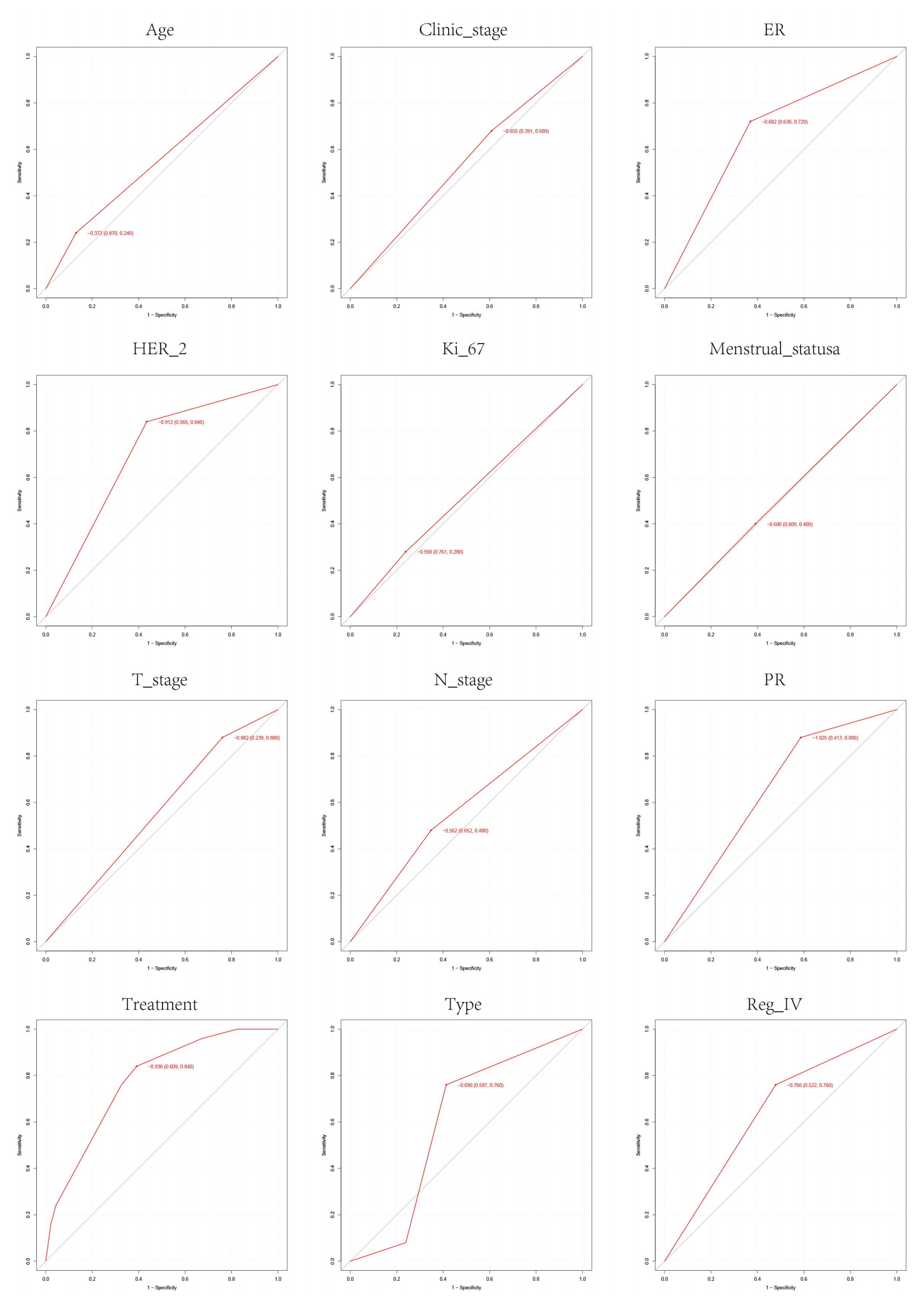

Supplement: Supplementary file 2 — ROC curves for each variable in univariate analysis for predicting pCR. Supplementary file2 (TIF 15112 KB) [file 12282_2024_1609_MOESM2_ESM.tif]
