# Supplementary material for: Development and validation of a neoadjuvant chemotherapy pathological complete remission model based on Reg IV expression in breast cancer tissues: a clinical retrospective study
Source: Breast Cancer. 2024 Jul 8;31(5):955–68. doi: 10.1007/s12282-024-01609-y (PMC11341653; doi:10.1007/s12282-024-01609-y)
Supplement: Supplementary file 3 — Supplementary file3 (DOCX 19 KB) [file 12282_2024_1609_MOESM3_ESM.docx]

Table S1 Baseline indexes

|  | **All data set** | **Train data set** | **Test data set** |
| --- | --- | --- | --- |
| **Characteristic** | **N = 104**^1^ | **N = 71**^1^ | **N = 33**^1^ |
| Treatment |  |  |  |
| AC | 3(2.9%) | 3(4.2%) | 0(0%) |
| AC-T | 16(15%) | 8(11%) | 8(24%) |
| AC-THP | 5(4.8%) | 5(7.0%) | 0(0%) |
| TAC | 25(24%) | 16(23%) | 9(27%) |
| TCbH | 2(1.9%) | 1(1.4%) | 1(3.0%) |
| TCbHP | 37(36%) | 26(37%) | 11(33%) |
| TCHP | 5(4.8%) | 3(4.2%) | 2(6.1%) |
| THP | 7(6.7%) | 5(7.0%) | 2(6.1%) |
| TP | 4(3.8%) | 4(5.6%) | 0(0%) |
| Age |  |  |  |
| <40 | 16(15%) | 12(17%) | 4(12%) |
| ≥40 | 88(85%) | 59(83%) | 29(88%) |
| Type |  |  |  |
| TNBC | 21(20%) | 13(18%) | 8(24%) |
| LUMI | 35(34%) | 25(35%) | 10(30%) |
| HER2 | 48(46%) | 33(46%) | 15(45%) |
| Clinic stage |  |  |  |
| T2 | 40(38%) | 26(37%) | 14(42%) |
| T3 | 64(62%) | 45(63%) | 19(58%) |
| Menstrual status |  |  |  |
| Premenopause | 41(39%) | 28(39%) | 13(39%) |
| Postmenopause | 63(61%) | 43(61%) | 20(61%) |
| T-stage |  |  |  |
| T2 | 79(76%) | 57(80%) | 22(67%) |
| T3 | 25(24%) | 14(20%) | 11(33%) |
| N-stage |  |  |  |
| Neg | 38(37%) | 28(39%) | 10(30%) |
| Pos | 66(63%) | 43(61%) | 23(70%) |
| ER |  |  |  |
| Neg | 50(48%) | 35(49%) | 15(45%) |
| Pos | 54(52%) | 36(51%) | 18(55%) |
| PR |  |  |  |
| Neg | 70(67%) | 49(69%) | 21(64%) |
| Pos | 34(33%) | 22(31%) | 12(36%) |
| HER-2 |  |  |  |
| Neg | 47(45%) | 30(42%) | 17(52%) |
| Pos | 57(55%) | 41(58%) | 16(48%) |
| Ki-67 |  |  |  |
| <20 | 23(22%) | 18(25%) | 5(15%) |
| ≥20 | 81(78%) | 53(75%) | 28(85%) |
| Reg IV |  |  |  |
| Neg | 44(42%) | 30(42%) | 14(42%) |
| Pos | 60(58%) | 41(58%) | 19(58%) |
| PCR |  |  |  |
| NonPCR | 67(64%) | 46(65%) | 21(64%) |
| PCR | 37(36%) | 25(35%) | 12(36%) |
| ^1^n(%) | | | |
